# Supplementary material for: Amplitude-Integrated EEG Monitoring in Pediatric Intensive Care: Prognostic Value in Meningitis before One Year of Age
Source: Children (Basel). 2022 May 5;9(5):668. doi: 10.3390/children9050668 (PMC9140190; doi:10.3390/children9050668)
Supplement: Supplementary file 1 [file children-09-00668-s001.zip › children-1692317-supplementary.pdf]

Supplementary materials

Table S1. PCPC score

Clinical Features	Category	Score
Normal at age-appropriate level<br>School age child attends regular school classroom	normal	1
Conscious alert and able to interact at an age-appropriate level<br>School age child attending regular school classroom but grade perhaps not appropriate for age<br>May have a mild neurologic deficit	mild disability	2
Conscious<br>Sufficient cerebral function for age-appropriate independent activities of daily life<br>School age child attending special education classroom<br>May have learning deficit	moderate disability	3
Conscious<br>Dependent on others for daily support because of impaired brain function	severe disability	4
Any degree of coma without any of the criteria for brain death<br>Unawareness even if awake in appearance without interaction with the environment<br>cerebral unresponsiveness<br>No evidence of cortical function and not aroused by verbal stimuli<br>Possibly some reflexive responses spontaneous eye opening and/or sleep-wake cycles	coma or vegetative state	5
Apnea OR<br>Areflexia OR<br>Electroencephalographic (EEG) silence	brain death	6

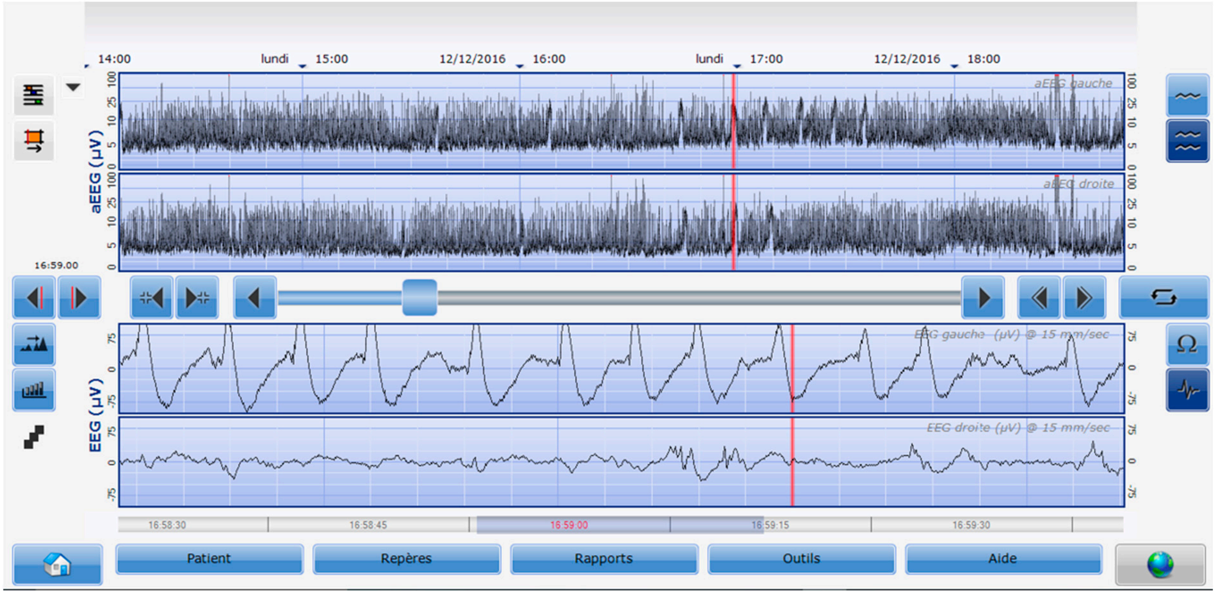

Figure S1. Example of seizure on aEEG trace.

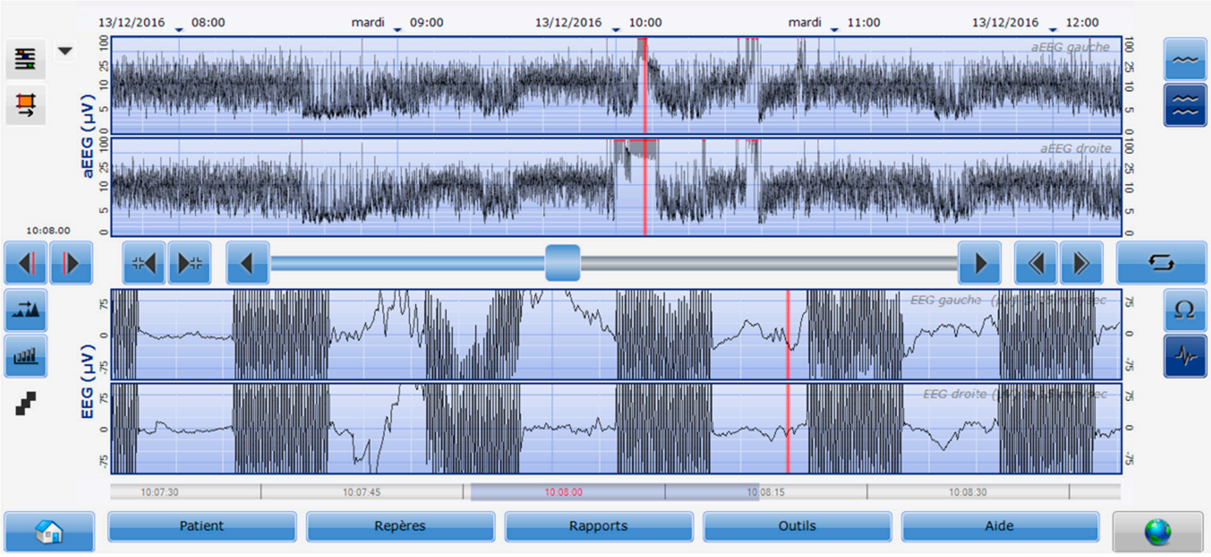

Figure S2. Example of a ventilation artifact.
